# Implementation of a decentralized community-based treatment program to improve the management of Buruli ulcer in the Ouinhi district of Benin, West Africa

**DOI:** 10.1371/journal.pntd.0006291

**Published:** 2018-03-12

**Authors:** Arnaud Setondji Amoussouhoui, Ghislain Emmanuel Sopoh, Anita Carolle Wadagni, Roch Christian Johnson, Paulin Aoulou, Inès Elvire Agbo, Jean-Gabin Houezo, Micah Boyer, Mark Nichter

**Affiliations:** 1 Buruli ulcer Treatment Center, Allada, Benin; 2 Regional Institute of Public Health of Ouidah, University of Abomey Calavi, Atlantique, Benin; 3 Centre Interfacultaire de Formation et de Recherche en Environnement et Développement Durable, University of Abomey Calavi, Atlantique, Benin; 4 Program against Buruli ulcer, Health Ministry, Cotonou, Littoral, Benin; 5 Anthropology department, University of South Florida, Tampa, South Florida, United Sates of America; 6 School of Anthropology, University of Arizona, Tucson, Arizona, United States of America; Swiss Tropical and Public Health Institute, SWITZERLAND

## Abstract

**Background:**

*Mycobacterium ulcerans* infection, commonly known as Buruli ulcer (BU), is a debilitating neglected tropical disease. Its management remains complex and has three main components: antibiotic treatment combining rifampicin and streptomycin for 56 days, wound dressings and skin grafts for large ulcerations, and physical therapy to prevent functional limitations after care. In Benin, BU patient care is being integrated into the government health system. In this paper, we report on an innovative pilot program designed to introduce BU decentralization in Ouinhi district, one of Benin’s most endemic districts previously served by centralized hospital-based care.

**Methodology/Principal findings:**

We conducted intervention-oriented research implemented in four steps: baseline study, training of health district clinical staff, outreach education, outcome and impact assessments. Study results demonstrated that early BU lesions (71% of all detected cases) could be treated in the community following outreach education, and that most of the afflicted were willing to accept decentralized treatment. Ninety-three percent were successfully treated with antibiotics alone. The impact evaluation found that community confidence in decentralized BU care was greatly enhanced by clinic staff who came to be seen as having expertise in the care of most chronic wounds.

**Conclusions/Significance:**

This study documents a successful BU outreach and decentralized care program reaching early BU cases not previously treated by a proactive centralized BU program. The pilot program further demonstrates the added value of integrated wound management for NTD control.

## Author summary

The management of Buruli ulcer (BU) is complex, resulting in high costs to families and health systems. Early detection and treatment heals lesions without functional limitations. Decentralization of the management of this disease into the peripheral health system remains a challenge for national control programs. We report here on an innovative pilot intervention of decentralization of the management of BU in one of the most BU-endemic districts in Benin. The intervention was preceded by a culturally sensitive outreach campaign, which explained the disease’s symptoms and treatment options, and increased self-referral. It also included the treatment of all chronic ulcers free of charge. While serious cases of BU were still referred to the reference hospital for treatment, study results showed that 71% of BU cases could be treated in decentralized care; of these, 93% were successfully treated without functional limitations with antibiotics alone. The decentralized treatment option brought in new cases not previously treated by a proactive centralized BU program, and maintained total patient adherence to treatment protocols, in part through the support of community volunteer groups. The model developed in this pilot study may serve as the foundation and proof of concept for a larger community-based decentralized wound care agenda.

## Introduction

Buruli ulcer (BU) is a debilitating neglected tropical disease caused by *Mycobacterium ulcerans*. It usually manifests through non-ulcerated lesions such as nodules, plaques, or edema, which may evolve into massive skin ulcerations, joint and bone deterioration if left untreated [1]. Most cases of BU are found in West Africa, and Benin is one of the most endemic countries [2]. Up until 13 years ago, the management of BU was surgical removal of all sites of infection. In 2004, antibiotic treatment was found effective at early stages of the disease (Category I: lesions < 5 cm in diameter; and Category II: lesions between 5 and 15 cm in diameter) [3]. At present, the management of BU has three main components: antibiotic treatment, surgical intervention, and physical therapy. Antibiotic treatment is based on daily oral rifampicin (10 mg/kg) and streptomycin (15 mg/Kg) injection for 56 days, which allows lesions whose diameter is less than 10 cm to heal without surgery [4, 5]. Effective antibiotic treatment at early stages thereby reduces wound dressings and avoids skin grafts, which are needed for large ulcerations. More advanced cases also require physical therapy to prevent disability, amputation, and functional limitations after care [6].

The National Control Program for Buruli ulcer in Benin supports four reference centers (CDTUBs) located in Allada (Atlantic department), Lalo (Couffo department), Pobè (Ouémé department) and Zangnanando (Zou department) for the detection and treatment of the disease. After five years of implementing a vertical approach to BU control that relied on surgery and hospitalization, BU patient care began being integrated into the government health system in 2004 for all but Category III cases (the most serious cases), which require surgery and are referred to reference centers.

The decentralization of BU care has several advantages. It increases community access to patient care and the chances of identifying BU cases early, and decreases the cost of treatment for the health care system and the burden on patients’ households, which have to bear indirect and opportunity costs even when free or subsidized treatment is offered [7]. Decentralized management of BU patients is now available in the Atlantic, Ouémé and Couffo regions of Benin and has resulted in improved early detection of BU [8]. The decentralized program has yet to be introduced in the Zou department, an area with a high prevalence of Category II and III BU cases. BU patients in this region are served almost entirely by a Catholic mission hospital which has become renowned for surgical treatment for BU.

In this paper, we report on the experience of introducing a BU decentralization pilot project in the Ouinhi district of the Zou department, a highly endemic area with many cases of patient delay despite the proactive efforts of a mission hospital to identify and treat all BU cases for free. The project was innovative in two notable ways. First, it introduced a recently developed, culturally sensitive approach to BU outreach education as a means of both raising consciousness about early stages of BU and informing the public about the availability of decentralized treatment. A second way it was innovative was its focus: the treatment and referral of all chronic ulcers, and not just BU, at local clinics following staff training in wound care. This decision to expand the scope of treatment was based on three factors: a recognized need to upgrade wound care skills in local clinics; the need to manage chronic wounds locally to reduce referral to busy hospitals; and community perceptions of the BU program as identified by social science research, which found that community members were confused about the differential treatment of wounds that appeared to them to be similar. They questioned why some wounds were treated free of cost, while other wounds were only treated if the patient paid for medicines and bandaging. Favoritism was suspected, and rumors to this effect undermined efforts to actively involve the community in BU identification.

Eight research questions guide the project. First, if we introduce a culturally sensitive outreach program that focuses on the early recognition of BU, and then offer decentralized care, does this result in treatment of BU cases not treated under the previous program, even in a region with a hospital proactive in BU case finding and with a strong reputation for centralized care? Second, how will the community respond to the innovative education program, and will it lead to cases of self-referral? Third, will the community accept decentralized care and adhere to the antibiotic protocols? Fourth, how will decentralized care and the outreach program affect women’s health care seeking behavior? Fifth, does the creation of social support groups add value for case detection, patient treatment adherence related to psychosocial support, and volunteer motivation? Sixth, will the treatment of all chronic ulcers at local clinics increase the reputation of the clinic and community satisfaction with the BU decentralized care program? Seventh, how will clinic staff respond to an expanded scope of wound care management after training and what will be the impacts of better feedback to clinicians regarding the accuracy and outcome of their referrals? And eighth, can a good working relationship between the decentralized and centralized BU care programs be established? The project was viewed as a feasibility (proof of concept) phase of a larger community-based wound care agenda that at once serves the needs of Benin’s Neglected Tropical Disease (NTD) program and the local need for community wound care education as basic to primary care.

### Study site and research background

**Fig 1:**
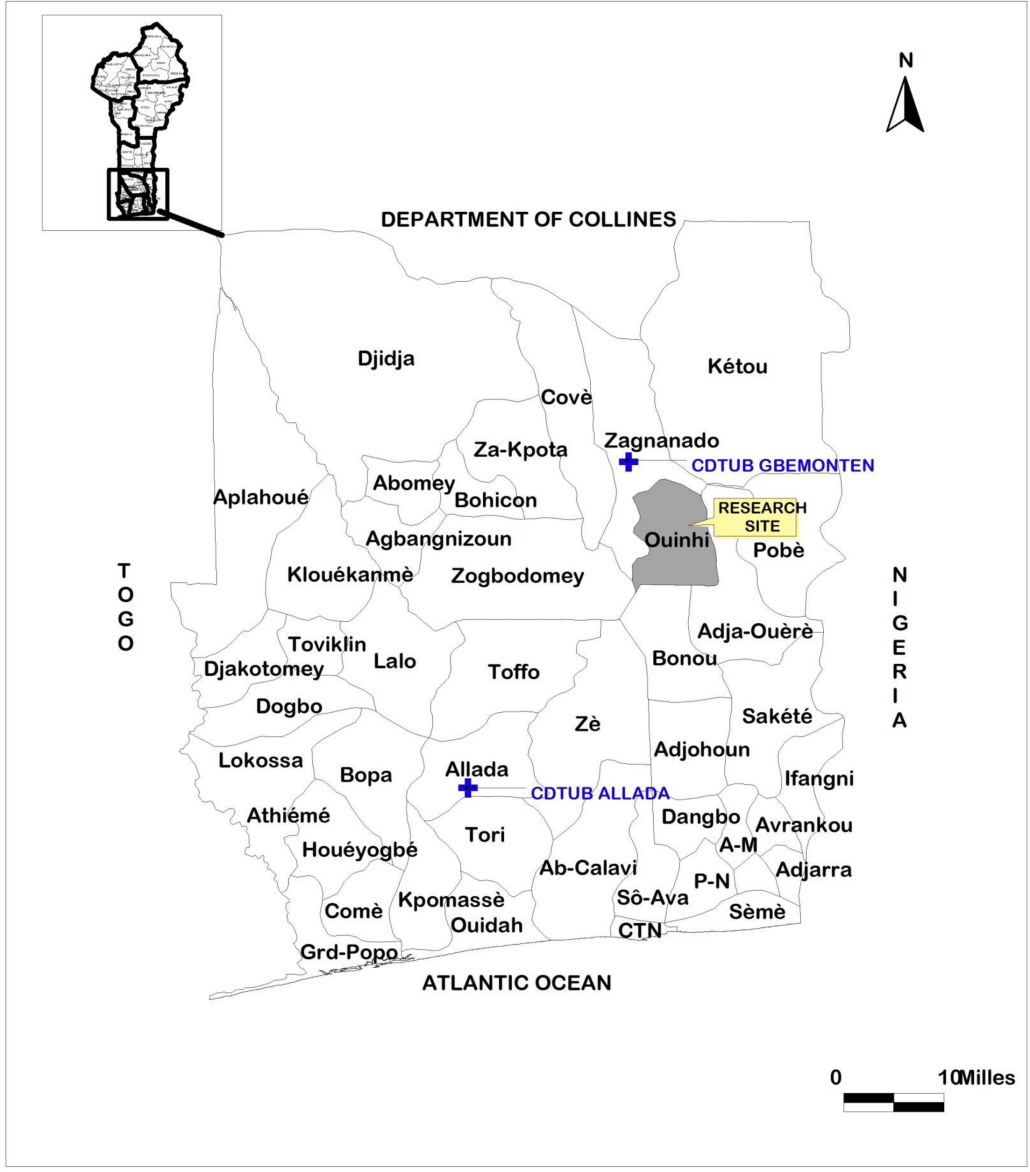
Location of the research site (ouinhi region), the CNSG of Zangnanado and the Buruli ulcer treatment center (CDTUB) of Allada in the south of Bénin.

**Fig 2:**
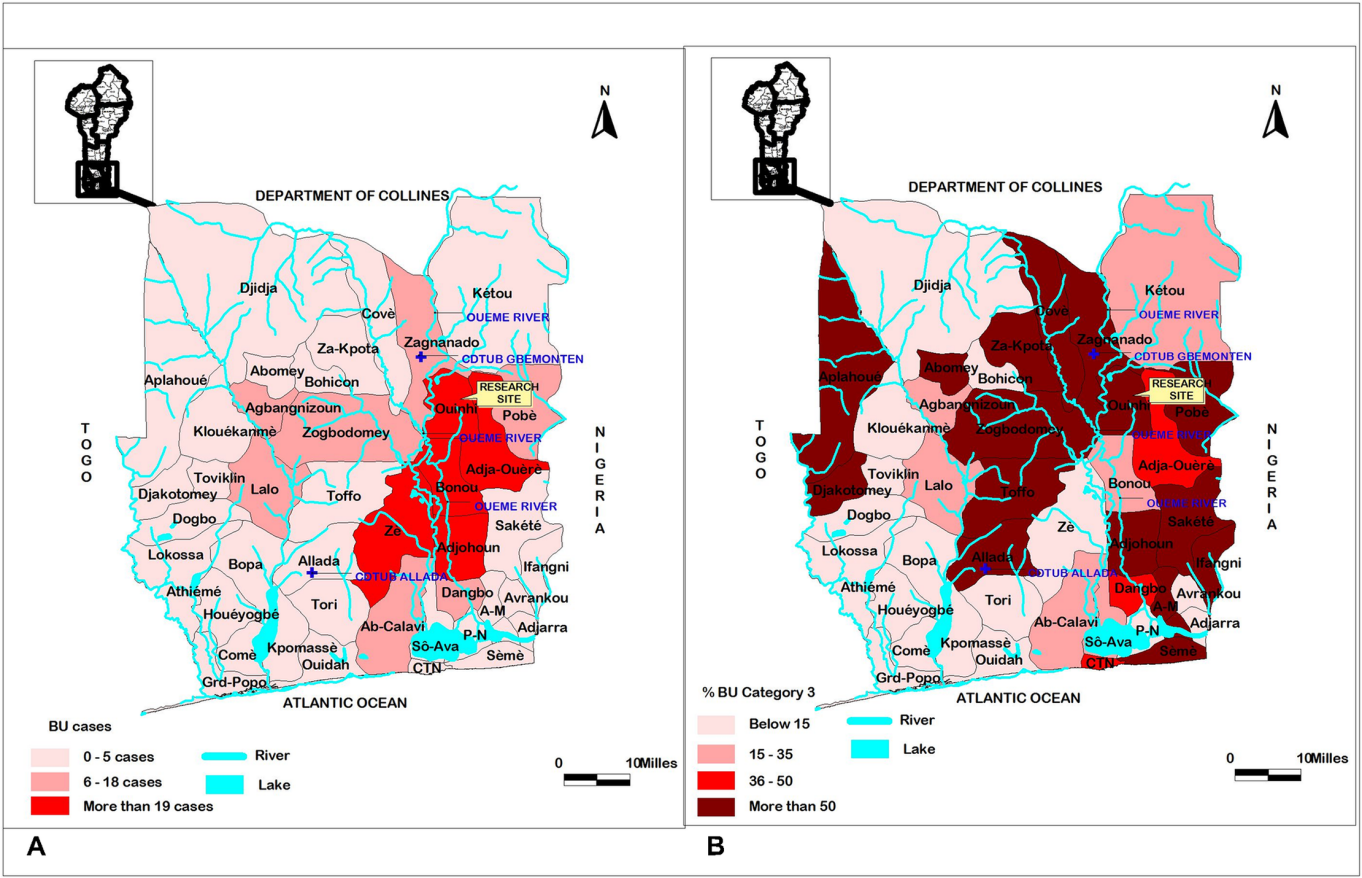
Endemic regions located in the south of Bénin (data source: PNLLUB, 2016 report). The five most endemic regions are located around the Ouèmè river (A); Ouinhi is one of the most endemic regions, showing the higher percentage of Category III cases (B).

The endemic Ouinhi district is located along the Ouémé River in southern Benin (Fig 1). It is one of the five most endemic districts (Fig 2A); of these, it has the highest number of Category III cases (Fig 2B).

**Fig 3:**
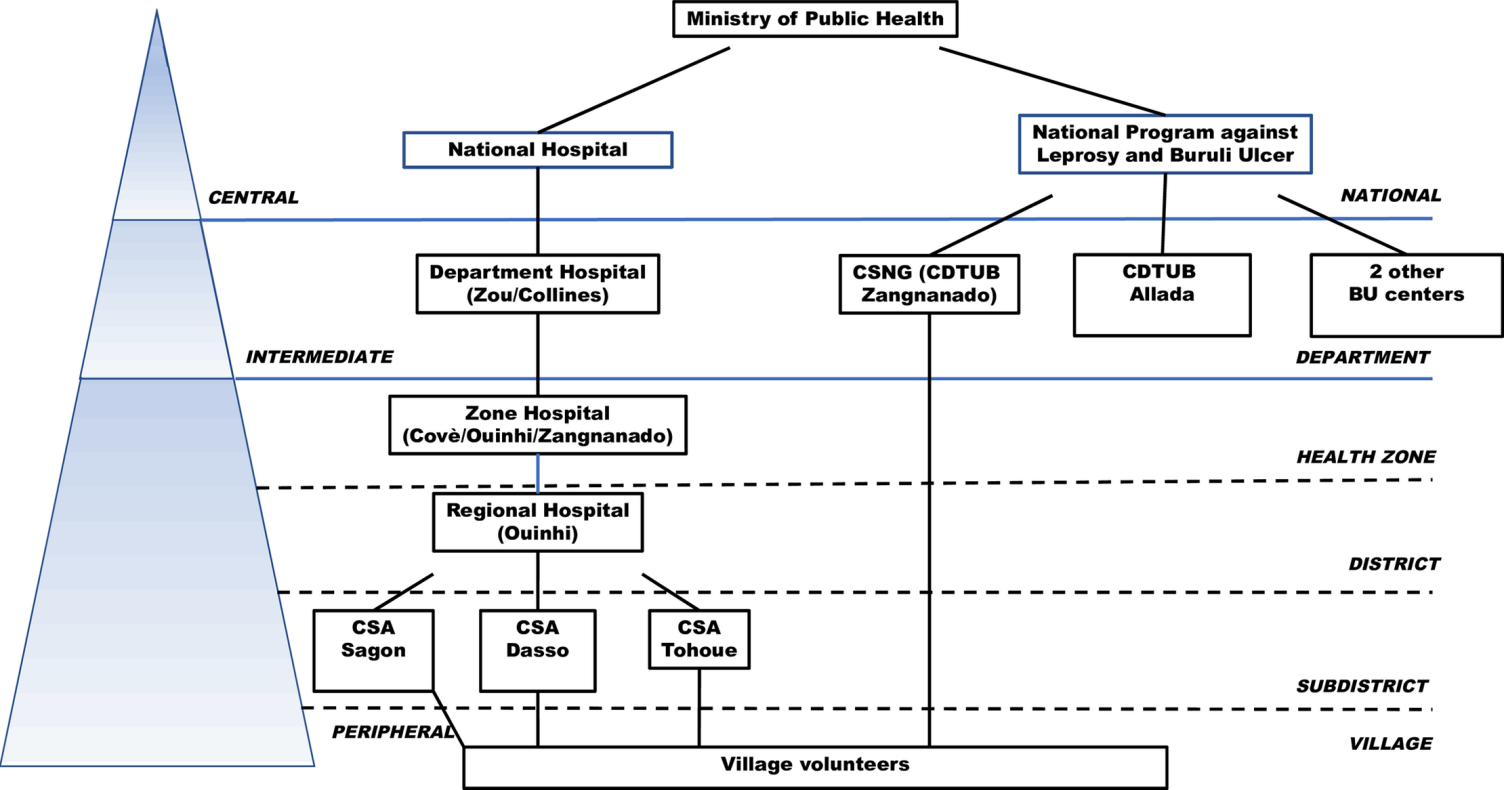
Organizational diagram of Benin health services to the intervention zone.

The local population depends on multiple household livelihood strategies, predominantly fishing, small-scale agriculture, and travel to urban areas in Benin and Nigeria for seasonal labor and remittance. Underdeveloped road and communication infrastructure limit community access to health resources. The research area is served by three local health centers in Sagon, Dasso, and Tohoue, which operate under the direction of the district hospital in Ouinhi (Fig 3).

Treatment modalities available in the region are diverse, and include: local clinics; drug peddlers in local markets and small pharmacies; traditional wound specialists who specialize in plant-based topical treatments or physiotherapy; and healers, diviners, and religious leaders that apply metaphysical techniques. There are multiple religions in the region including Islam, Catholicism, Pentecostal Christianity, and *vodun*, the indigenous religion of the predominant ethnic group in the region, the Mahi-Fon. The diversity of medical and religious practices leaves the population open to multiple possible interpretations of chronic wounds that do not heal.

**Fig 4:**
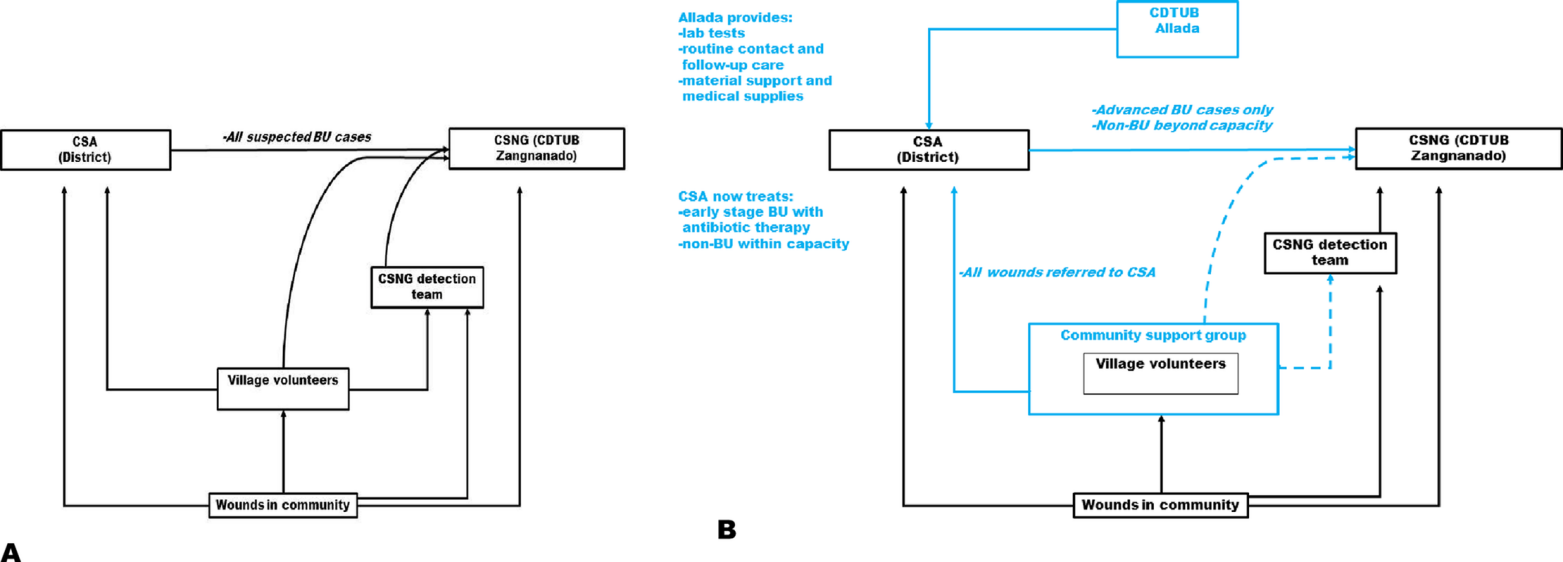
Referral and treatment system before (A) and after (B) the introduction of the pilot intervention.

Previous research [9–11] found that households often seek multiple forms of health care simultaneously, and may add any of the above modalities to biomedical care. Before the pilot intervention, suspected cases of BU either went to clinics in the national health system or were brought directly to the nearest center specialized in Buruli ulcer treatment, the *Centre Sanitaire et Nutritionnel Gbemoten* (CSNG) (Fig 4A).

Located in Zangnanado, CSNG is one of the four reference centers for BU treatment (CDTUBs) that form the national BU system (Fig 3). Operated as a private Catholic facility, Zangnanado is relatively close (10–15 miles) to most patients in the study area, although accessibility becomes an issue during the rainy season, when much of the endemic region is flooded. CSNG covers much of the cost of BU treatment and transportation to the hospital, and has established a vigilant system of case detection, since the hospital often faces resistance from suspected cases. CSNG has been treating Buruli ulcer in Benin since 1989, and has developed considerable surgical expertise in its treatment and an exceptionally low rate of relapse [12, 13].

The center has developed an extensive network of village volunteers who work in collaboration with trained health workers to identify cases and raise awareness about the disease. Before decentralization of Buruli ulcer care, clinics in the endemic region collaborated with CSNG staff to refer all suspected BU cases (Fig 4A), following a general protocol of excision even for Category I ulcers.

A key factor in getting CSNG to accept the pilot was the inclusion of other wounds in the decentralization protocols. Because tropical ulcers and other chronic wounds are often unresponsive to treatment and can require extensive stays, hospitals are obliged to expend considerable financial and human resources in their treatment. The new protocols promised to reduce the burden of treating chronic ulcers in the hospital by catching them early enough to be treated at local clinics. This would allow CSNG staff to focus their resources on their area of expertise: surgical care of advanced Buruli cases.

The pilot intervention also required the collaboration of officials of the national health system. Permission was granted by both the coordinator of the zone hospital in Covè and the head doctor of the district hospital in Ouinhi who oversees the clinics under his jurisdiction. The current head district doctor was particularly receptive to the idea of introducing decentralized care in the clinics.

## Methods

A review of the national program against BU’s epidemiological data identified Ouinhi as the most endemic district for BU of the five covered by CSNG. Thirty-eight percent of all BU patients treated in Benin live in this district. Our action-oriented research was conducted in four steps: baseline study, outreach education, training of Ouinhi health clinical staff, and outcome and impact assessments. The overall intervention was supported by the establishment of community BU support groups and social assistance to vulnerable patients. In the present case, decentralized BU treatment had yet to be established.

### Ethics statement

As part of a community intervention, this study was approved by the Benin’s National Ethical Committee for Health research (IRB00006860 N°148/MS/DC/SGM/DFRS/CNPERS/SA). The national and regional health authorities also gave their authorization for the implementation of the study and fully participated in the interventions. All participants (patients, village volunteers and local staffs) were widely informed about the study and the process. They were assured about the anonymity of all data collected and any information gathered during the evaluation process. All patients as well as interviewees signed a written informed consent form, after the content had been fully explained to them by a local translator independent of the team of researchers (for those who were illiterate). For participants under 18 years old, they were informed together with their parent (usually the mother) or guardian before the parent / guardian signed the consent form. A specific informed consent form was given by the Tohoue nurse for the inclusion of his photograph in the article.

### Baseline study

A baseline study of BU-related perceptions and healthcare-seeking behavior was conducted in 2011. The primary aim of the study was to identify determinants of treatment delay in the most endemic district covered by CSNG. One hundred and two households with patients treated for BU in areas covered by CSNG from 2000 to 2010 were interviewed. This first stage of formative research investigated: local perceptions of all chronic ulcers; recognition of the signs of BU at different stages; home-based wound care; healthcare-seeking behavior for chronic wounds; reasons for consulting (and not consulting) available biomedical facilities; and reasons for delaying treatment. Our research was informed by previous social science investigations of BU conducted in Benin [10] as well as elsewhere in West Africa [14,7,15,16]. The perceptions and experiences of former and current patients have proven to be valuable when designing both community outreach and clinic based BU interventions [17,18].

### Outreach education

**Fig 5:**
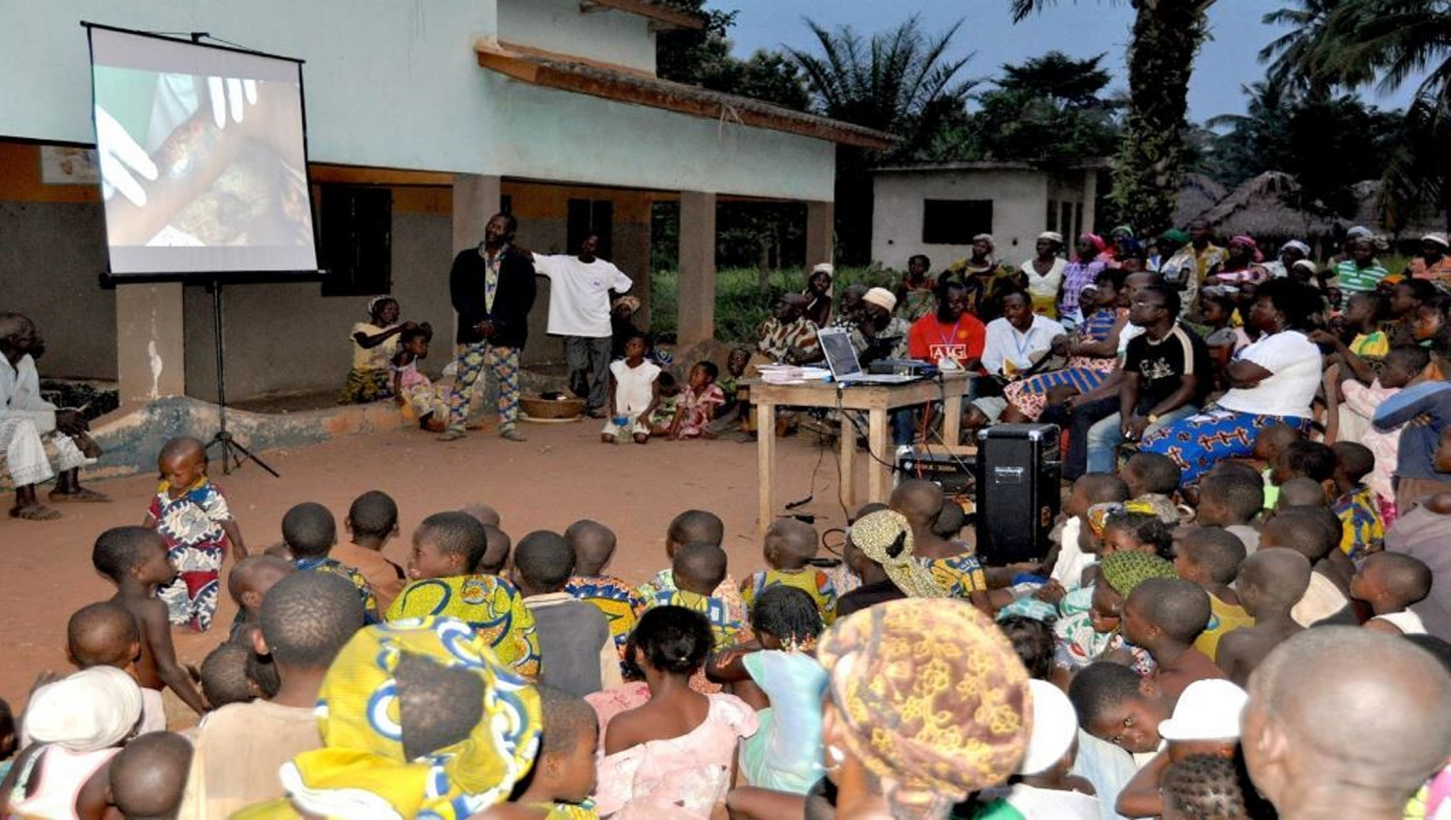
Awareness-raising session conducted in Tohoue by the study team with the help of village volunteers.

An innovative outreach education program being developed by the Stop Buruli Consortium in Benin, Cameroon, and Ghana [19] was adapted for use in Ouinhi based on the findings of stage one formative research. The education program took the form of an image-rich PowerPoint presentation about BU delivered by clinic staff and health volunteers equipped with generators, computers, LCD projectors and sound systems (Fig 5). A question–answer format was adopted with new questions added as they arose during community meetings (Table 1) as part of an iterative process. Mass outreach education events were designed to be interactive, not passive, and questions were invited from the community. PowerPoint presentations were employed because they are easy to modify in response to questions posed and issued raised. Social scientists conducted ongoing translational research to identify how best to respond to questions in a way that was at once scientifically accurate and comprehensible to local audiences. Messages and visuals were tested and changed as needed.

**Table 1:** Structure of the outreach education program.

Sections of outreach education program	Key messages conveyed	Issues downplayed or emphasized
Signs and symptoms of BU, how to recognize the disease and need to treat early	Visuals of physical signs of BU in different stages	Category I and II BU depicted, but not Category III as this evoked great fear.
	Visual and tactile cues suggesting that an abscess or ulcer may be BU	
	Progression of disease if not treated	
High risk environments and modes of transmission	High risk environments where one is more likely to be exposed to BU	Less time and attention was allotted to risk environments and possible modes of transmission as the science is inconclusive and behavior change related to exposure to water sources difficult given the local reality
	Attention was dedicated to addressing incorrect ideas about BU transmission and contagion	
What clinic staff do to determine if the affliction is BU or some other disease	Why health staff take swabs, what they look for under the microscope, why medicine for BU is specific and not the same as medications used for other ulcers.	Step-by-step explanation of what staff do along with pictures—- to offset fears and rumors about what health staff is doing, and to increase trust
Effective and ineffective treatments for BU	Why 56 days of pills and injections are needed	Agricultural analogies used to convey the idea that medication is taken beyond cure of wound to get at the roots and seeds of BU as a systemic infection in the body
	Why herbal medicine for this disease does not lead to a cure even if a wound is dried	
		Pictures used to show inappropriate treatment, how drying wound is not healing, and effectiveness of medication after herbal medicine has failed to treat the wound
Traditional healers and rapid referral to clinics	Positive messages about exemplar healers who recognize signs of BU and rapidly refer patients to clinic after spiritual protection is offered.	No message disrespecting local practices as superstitious.
Emphasis on rapid referral		
		Respect for traditional healers’ role in offering spiritual protection for those for whom this is a concern.
Quality of care at the clinic	Quality of care offered by staff: pictures of what care in the clinic looks like, approachable staff, hygienic conditions, empathetic guardians, etc.	To offset fear and evoke confidence
Before and after pictures of BU related wounds successfully treated	Pictures of BU treatment, and the healing process at different stages	Pictures depict patients of different ages and gender
	Depict the healing of ulcers on different parts of the body	
The presentation ends on a note of hope	Testimonials of patients who have been cured speaking of their experience and the quality of care they have received at the clinic.	Open microphone–some testimonials are planned and others are spontaneous
Questions from the audience	On any topic related to information presented or any other issue related to BU	Open microphone

Community outreach education meetings were held in the evenings, organized by local volunteers, and facilitated by volunteers and clinic staff trained in how to communicate information and field questions from the community. The social science team responsible for developing the educational program selected and mentored a CDTUB staff member with strong communication skills to serve as a health staff trainer. Prior to functioning as a trainer, he had several months of experience delivering education programs during the pretesting of the outreach program. Training was practical and focused on how to present the material on slides in an easy-to-understand manner, respond to common questions raised by the audience, and facilitate interaction. Social scientists monitored all outreach programs as a means of collecting community feedback to modify the educational program. Village chiefs, local healers, and former patients were invited to all outreach meetings.

At the conclusion of outreach meetings, health staff offered the opportunity for screening to those community members who wished to learn whether their wounds might be BU, either at that time or at a participating clinic the next day. BU community support groups were established in villages associated with each participating clinic to help identify possible BU cases, encourage all villagers with chronic ulcers or wounds to seek treatment at participating clinics, provide psychosocial support during the process of decentralized BU treatment, and to follow up on cases of treatment dropout. These groups were composed of village volunteers who had already been working with CSNG previously, health volunteers from other programs, village leaders, and healers and religious leaders interested in participating. Groups met at clinics once a month and coordinated activities with clinic staff.

### Community support groups and social assistance to vulnerable patients

In order to facilitate the introduction of decentralized treatment in Ouinhi, a support group was established in each district. Support groups included representatives from each village and were selected by village chiefs and their advisors. The proposed list was then confirmed with health staff from the district healthcare center, and three members were selected as officers (a chair, a secretary, and a treasurer). Support group members helped arrange and attended outreach activities, assisted in early detection, and identified vulnerable patients (for all wound types) in particular need of assistance. The assistance provided to those in dire need consisted of daily transport for treatment for those living more than 2 kilometers from a clinic, a modest food allowance, and school support for children who might otherwise drop out of school as a result of treatment. Support in the form of food and transportation have increased BU treatment acceptability and adherence in established decentralized treatment programs in countries such as Ghana [20].

### Staff training

Over the course of two months, the medical staff of CDTUB Allada trained Ouinhi district health personnel in the Center on the principles of wound care. The medical doctor responsible for Ouinhi district hospital, the nurses responsible for three clinics in Ouinhi, and two attendants per clinic all received training.

The training was conducted in six stages. First, the doctor from the district hospital was brought up to date on major research findings related to the epidemiology and treatment of Buruli ulcer. Emphasis was placed on advances in BU control and care since the setting of the Global Buruli ulcer initiative in 1997. Second, the two nurses and their clinic attendants were given a less intensive orientation into advances in BU research and treatment as well as the basic principles of wound care with an emphasis placed on wound hygiene. Instruction was also given on: how to diagnose BU; differential diagnosis of BU per type of lesion (nodule, plaque, ulcer); categorization of BU; the characteristics and differential diagnosis of common types of chronic ulcers and skin diseases; general principles of treatment and different components of BU treatment; reference criteria; identification and management of treatment side effects and contraindications such as during pregnancy; therapeutic management of cases (BU and leprosy, BU and pregnancy, BU and tuberculosis); different types of wound dressings and steps for each type; basic principles of wound dressing; when to request laboratory analysis; how to take swabs; and how to send samples for laboratory analysis. Third, existing health staff wound care practices were elicited, reviewed and compared against best practices advocated by WHO [21]. Fourth, practical clinical training was provided by staff at the Allada CDTUB. The practical training included: general wound hygiene techniques; bandaging skills; recognition and prevention of common wound infections; and basic physical therapy as a means of disability prevention. Trainees were provided hands-on experience with BU patients at Allada hospital. Fifth, trainees visited decentralized centers of Zè district and participated in active case detection. They also participated in BU outreach programs using the innovative approach to BU education being introduced in Ouinhi and first pretested in Zè. Sixth, trainees were instructed on how to complete wound reporting and treatment monitoring data sheets. Upon oral examination at the end of the training period, trainees were able to successfully demonstrate knowledge of the materials covered throughout the training, with particular focus on BU diagnosis criteria, the use of streptomycin and rifampicin, and protocols for wound care.

Following training, health staff returned to their clinics. Staff from Allada visited them every time they suspected a BU case. During their visit in Ouinhi, staff from Allada supervised and monitored practices of the trainees, and they were in routine contact with staff. When required, advanced BU patients were referred to CSNG. Other chronic ulcers (e.g., vascular ulcers, cancers, or large non-BU ulcers) requiring advanced care were referred to CSNG or another appropriate treatment center (Fig 4B).

Lab tests were requested for every suspected case of BU, and staff from Allada, assisted by the district health workers, collected the samples (FNA for non-ulcerated lesions and swabs for ulcers) required for laboratory analysis. Samples were sent to Allada CDTUB for direct smear examination and to the reference laboratory for mycobacteria in Cotonou, for PCR. Allada also provided medical supplies (antibiotics, wound dressing material, sample collection material) to clinics as needed.

### Outcome and impact assessments

Outcome and impact assessments of the project were conducted two years after the pilot intervention was initiated. The outcome assessment reviewed quantitative data on cases seen and successfully treated or referred. This entailed a review of clinic records on patient self-referral for wounds suggestive of different categories of BU as well as other chronic ulcers of at least six months’ duration. Data was also collected on patient adherence to decentralized BU treatment and the referral of cases from Tohoue to other clinical facilities. The impact evaluation entailed interviews and focus groups with clinic staff, patients, community volunteers and local leaders, and health officials. It generated in-depth qualitative data that considered intended and unintended effects of the intervention within the community. Interviews focused on: lines of communication and collaboration between clinic and community stakeholders; patient satisfaction with decentralized care; and the impact of expanded care on the reputation of the clinic and the status of health care workers and volunteers. The impact evaluation was conducted by a team of four social scientists and clinicians who conducted interviews with the informants listed in Table 2. All interviews were conducted using pretested interview guides.

**Table 2:** participants in impact evaluation interviews.

Persons interviewed	Number
Former patients	7
Members of BU support groups	25
Healers	9
Volunteers who facilitate meetings	4
Staff of decentralized clinic	4
Public Health Staff, Ouinhi district	3
Staff, Allada Hospital	2
Members National BU program (PNLLUB)	3

### Maps

The maps presented in this article (Figs 1 and 2) were drawn using QGIS 1.8.0 and ArcView 3.2 software, based on open access shapes files obtained from www.diva-gis.org.

## Results

### Baseline study

The baseline study revealed poor community-level recognition of Category I BU, but some level of familiarity with the signs of more advanced BU due to the outreach efforts of CSNG. Research revealed that enabling and health service-related factors delayed health care seeking for chronic wounds far more than predisposing factors related to such cultural concerns as witchcraft [cf. 22,23,24]. In households where suspected cases of BU had been identified, family members were often reluctant to accept the diagnosis, and had delayed hospital treatment due to practical concerns and rumors about what transpired within the hospital. These concerns included: competing work and child care obligations fundamental to household survival; concern about meeting indirect costs of hospital care; fear of abandonment; and fear of amputation (often described within the community as a standard practice at CSNG). Former patients also frequently complained of not being informed about how long their hospitalization would last and not being informed about how their treatment was progressing.

### Outreach education

Outreach education programs were carried out in 22 villages in the Ouinhi health district. Approximately 4000 community members attended outreach meetings over the course of two years, with an audience size of 140–300 people per outreach program. Village leaders and local healers who supported BU outreach activities attended all meetings and contributed to their legitimacy. Half of the 20 outreach sessions were led by the staff nurse of Tohoue clinic, and the other half by village volunteers who had proven to be effective communicators in past health programs. Among these volunteers, one in particular was looked to for guidance, and he assisted other volunteers from several other villages in organizing and conducting outreach programs.

Post-outreach interviews with 60 key informants from 15 villages conducted the day after outreach found that community members greatly appreciated the question and answer framework that provided the structure for the educational PowerPoint. Community members also appreciated the use of images to depict key messages, before and after pictures of wounds that had been treated, and the testimonials of past patients. The testimonials offered at outreach meetings were initially presented by former patients treated in centralized care who had recovered. As the project progressed, patients successfully treated in decentralized care began providing testimonials as well. The community was very happy to hear that free outpatient treatment was available for not just BU, but for all chronic wounds at Tohoue, and they felt reassured when patients spoke of their positive experience with health staff.

### Staff training

The staff of the three healthcare centers selected in the Ouinhi district already had basic knowledge of Buruli ulcer prior to the training, but were not aware of decentralized treatment protocols or their effectiveness, and did not feel competent to treat BU or other chronic ulcers. Chronic ulcers were referred and not treated in their clinics prior to training. Training addressed knowledge gaps in wound assessment and wound care procedures, especially antibiotic treatment protocols. After the training period in Allada, they were able to make a more accurate clinical diagnosis of BU, provide and follow appropriate antibiotic treatment, and deliver correct wound care to patients. Training also taught staff to provide adequate wound care to chronic non-complicated ulcers, thereby avoiding the systematic referral of simple cases to CSNG.

### Referral, treatment, and adherence outcomes

Table 3 summarizes patterns of referral, treatment and adherence to treatment in the Ouinhi district following the pilot intervention. A few findings are worth highlighting. Ouinhi is a district in which proactive BU case finding has been going on for over two decades by CSNG hospital. CSNG is highly motivated to identify BU cases inasmuch as its funding is tied to treating BU surgically. While the hospital has an impressive track record of treating more serious Category II and III BU cases, its record for detecting Category I cases is well below the national average. The pilot study demonstrated that Category I cases could be identified in the community following outreach education, and that these community members were willing to accept decentralized treatment. During the impact evaluation, the families of several successfully treated patients under the decentralized scheme reported that they had refused to be treated at CSNG or had delayed doing so because of grave concerns as to how this would affect the well-being of their household.

**Table 3:** Buruli (BU) and chronic (CU) ulcers detected following outreach programs.

Health Center Location		Tohoue	Dasso	Total
All cases after outreach		85	11	96
Suspected BU cases		46	7	53
Confirmed cases	Total–n (%)	37 (77%)	4 (57%)	41 (77%)
	Category I or II	27	2	29
	Category III	10	2	12*
Category I and II treated locally		29**	0**	29 (100%)
CU (not BU) brought to clinics		48	0	48
CU treated locally		14 (29%)***	0	14 (29%)

* All Category III cases were referred directly to CSNG.

** The two confirmed early-stage cases from Dasso came to Tohoue for decentralized treatment

*** Three of these cases came from Dasso

Outreach education increased BU identification by community members. In the two years prior to the pilot project, the nurse at Tohoue clinic saw 14 chronic ulcer patients (Buruli ulcer and other ulcers). All of these cases were referred to CSNG, and he received no feedback about them. After the outreach program, the number of community members with chronic ulcers coming to the Tohoue and Dasso clinics soared to 96 (over a two-year period of time), with most patients coming directly to the Tohoue clinic because of the reputation of the nurse for treating cases effectively. A smaller number of chronic ulcer cases visited Dasso clinic located 5 kilometers away, but treatment was not offered. Fifty-three of the 96 cases of chronic ulcer (55%) who were seen at Tohoue and Dasso were self-referred, with the remaining 43 (45%) referred by support group members. During the impact evaluation, the families of several successfully treated patients under the new decentralized scheme described how outreach programs had convinced them of the need for early treatment. Following outreach programs, they had sought care at the local clinic without delay.

Notably, of the 96 chronic ulcer cases seen in Tohoue and Dasso, 53 (55%) were suspected to be BU cases, and of these 41 (77%) were confirmed to be BU. Of these confirmed cases 29 (71%) were Category I or II BU cases treatable by a decentralized care protocol (Picture 3A and 3B). Impressively, all BU cases treated completed a full 56 days of treatment. Twenty-seven of the 29 cases of BU in decentralized care (93%) were successfully treated at Tohoue. The two remaining cases eventually required surgery and were referred to CSNG for surgery. Another 48 cases of chronic ulcer were seen at the clinic, of which 14 were treated. The remaining 34 cases were beyond the capability of the nurse to treat and were referred to CSNG for surgery.

### Community support groups and social assistance to vulnerable patients

Out of the 96 chronic ulcer patients treated by the two healthcare centers, 43 were referred by support group members and of these 27 were suspected BU cases. Notably, traditional healers referred 7 of these patients. Nineteen out of the 27 suspected BU cases proved positive for the PCR and 11 patients were followed up for treatment compliance by the support group.

As part of the social assistance provided to vulnerable patients, daily transport to the clinic and feeding were provided respectively to 10 patients and 35 patients. Transport to CSNG was provided for 35 patients, including both advanced (Category III) BU cases and non-BU chronic ulcers. Medication assistance was offered to 22 non-BU patients, and 8 children were offered support to keep them in school.

### Shifting roles and relationships for the reference centers

CSNG, the regional reference center for Buruli ulcer, has experienced three major shifts in responsibilities as a result of the intervention. First, patients in the Ouinhi district now have the choice to seek decentralized, non-surgical care at peripheral health clinics for early-stage Buruli ulcers rather than additional surgical care at CSNG for all BU cases. Second, the care of chronic, non-Buruli ulcers is now offered by the clinics participating in the pilot project. Finally, CDTUB Allada, the country’s main reference center for Buruli ulcer, oversees the follow-up care for wounds treated in the peripheral clinics. The impact evaluation found that while serious cases of BU are being referred to CSNG hospital, CSNG is not referring non-BU cases to Tohoue clinic for treatment or to manage postsurgical follow-up care on an outpatient basis. There was one exception. The parents of a girl whose Buruli ulcer had not responded well to repeated surgeries at CSNG and required further treatment was told by staff that they might try decentralized treatment with antibiotics at Tohoue clinic during the month that CSNG was closed. The patient did so with a positive result.

CSNG’s participation in the project has thus far been a peaceful coexistence with the decentralized BU pilot. Allada and CSNG have maintained a strong professional relationship, and in the future CSNG will presumably benefit from a reduced number of non-BU chronic ulcers to better focus its considerable expertise on the treatment of advanced Buruli cases. If the decentralized pilot goes to scale, and each clinic in the district is able to treat the same number of cases as Tohoue, the burden on CSNG will be reduced significantly. CSNG’s proactive outreach system remains in place in Ouinhi, and the two systems work in parallel providing greater and more effective coverage for BU case detection.

### Shifting roles and relationships for the peripheral clinics

**Fig 6:**
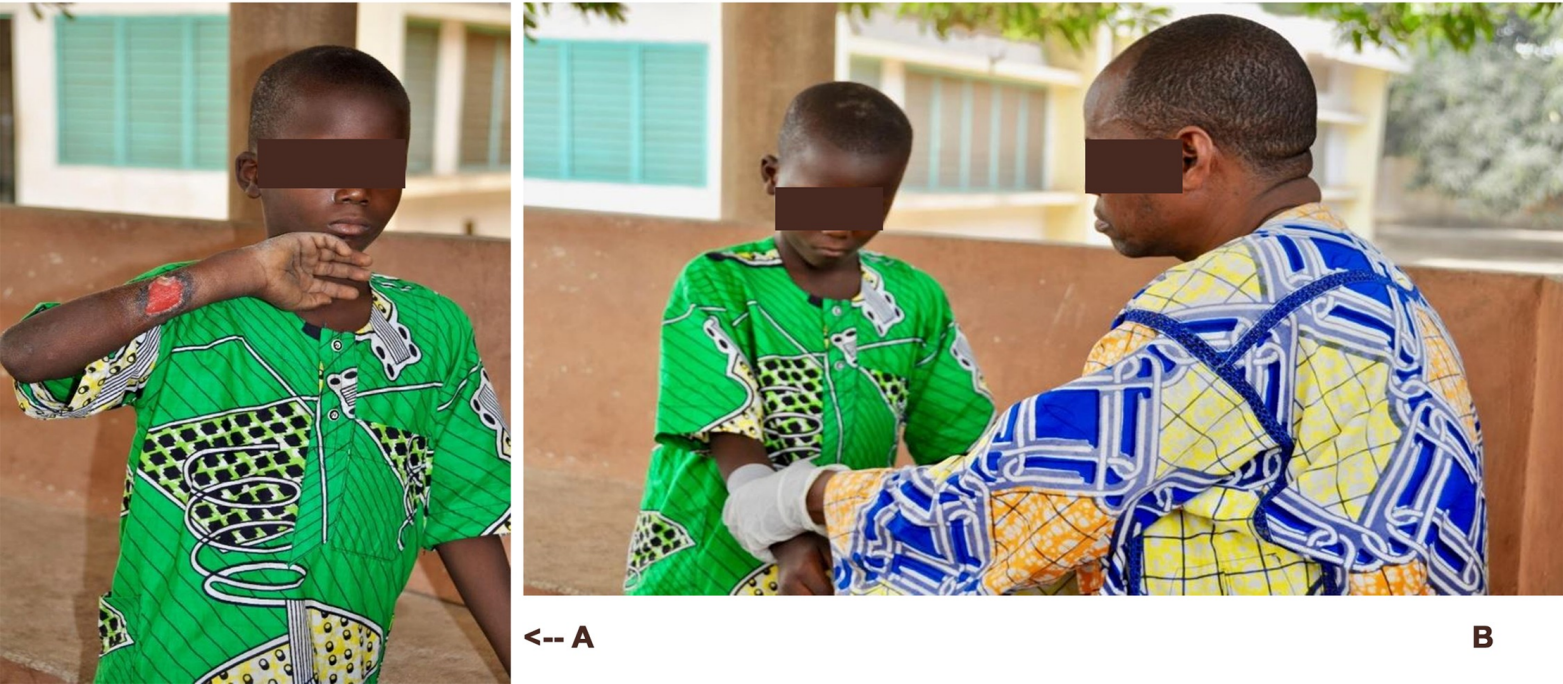
Child diagnosed with a Category II suspected BU lesion (A), examined by the Tohoue health center nurse (B).

The clinic at Tohoue has assumed the care of early BU cases, and with the expertise and material support of the Allada reference center, has also accepted care of chronic ulcers within their capacity to treat. The impact evaluation found that this shift in the focus of wound care has resulted in an increase in social capital for the clinic, but also an increase in duties and responsibilities of staff without commensurate increases in salary. The nurse who directs the Tohoue clinic (Fig 6) was already very popular, and participation in the pilot project has increased his reputation in the community in two ways. As the presenter in approximately half of all outreach programs, his visibility and reputation for wound care expertise has grown and been reinforced. His collaboration with the Allada CDTUB and increased ability to triage chronic ulcer cases when necessary has also signaled to the community that he has access to a broader health care network and resources.

For example, the nurse agreed to treat a nine-year-old boy who was bitten by a snake and who had been told by doctors at two hospitals that his leg would have to be amputated. The family refused to accept amputation and upon hearing about free care at Tohoue consulted the nurse. The nurse treated the wound, the boy kept his leg, and the nurse was able to arrange for the boy to receive physical therapy at Allada. At a time when many local clinics are chronically under-resourced, having access to adequate supplies for ulcer management and being able to personally refer cases of wounds requiring advanced care is a powerful form of capital when serving a population that deals with uncertainty at nearly all levels of health-seeking.

The success of the pilot project in treating all cases of Category I and II BU, without incurring the costs of centralized care to households, provides a powerful demonstration effect for the larger community of the viability of decentralized care, the treatment protocol’s effectiveness, and Tohoue staff’s clinical competence. The evaluation found evidence that greater respect for Tohoue clinic (as a source of reliable, professional, and empathetic care) has had an impact beyond wound management. The reputation of the clinic is now being leveraged for other community-based health care activities.

Given these advantages, the nurse and his clinic attendants remain supportive of the intervention, although they acknowledge that it has increased their workload in an already understaffed clinic. Moreover, decentralized care, its supervision, and the treatment of other chronic wounds incurred additional costs as did patient support in the form of feeding. In order to sustain clinic success these costs would need to be covered in the future. More pressing was another point of concern. Raising community consciousness about the importance of wound care has the potential of indirectly encouraging people to visit the clinic with wounds that might be managed at home. To reduce the burden on clinics like Tohoue, and the health care system in general, practical community-based wound care education will be necessary. Wound care education will need to focus on common wounds and skin diseases as well as follow-up care and scar management for chronic ulcers.

### Volunteers and the added value of BU support groups

Village volunteer networks serve as an invaluable if under-acknowledged resource for the health system, providing a level of surveillance and contact that neither the national health system nor the detection team from a reference hospital can match. As a result of the intervention, village volunteers saw several significant modifications to their roles: they shared in the work of detection with other members of the newly-formed community support groups; they accompanied suspected early BU cases to a local clinic; and once a patient was treated at a clinic, they provided ongoing psychosocial support. The impact evaluation found that psychosocial support provided by support group members contributed to the 100% adherence rate of decentralized BU patients. The impact evaluation also found that volunteers now interacted in positive ways with healers and religious leaders participating in support groups and their collaboration was seen by the community in a positive light.

### The participation of traditional healers

The intervention provided an opportunity for traditional healers and religious leaders to participate in support groups and be advocates for decentralized BU treatment. Several local healers and religious leaders became members of support groups and 16 healers actively participated in outreach programs. At the beginning of the project some concern was raised about allowing healers to participate in support groups based on negative experiences in the past. A few years before the pilot intervention, two healers who attended an NTD training used course attendance as a means of recruiting and keeping patients instead of referring them. The impact evaluation found no case where a healer used membership in a support group to claim expertise in curing chronic ulcers or as a means of attracting new clients. On the contrary, their attendance at outreach programs demonstrated to the public that they advocated promptly taking suspected cases of BU to local clinics. Other support group members spoke of the cultural sensitivity involved in negotiating the transfer of patients from traditional to biomedical care. A 29-year-old community support group member noted:

“We understand our responsibilities and our limitations. We’ve had no problems at all with traditional healers as long as we don’t interfere with their livelihood. If we find out that there is a potential Buruli ulcer case in treatment at a healer’s home, we don’t speak to the healer, but go through the relatives of the patient to convince them to get him treatment at the health center. That’s what happened with a 17-year-old girl who was staying at a healer’s home. One of her relatives had attended an outreach event, and we contacted him afterwards and communicated back and forth, presenting the advantages of following treatment in the peripheral health centers. Through the relative’s complicity, we were able to convince the girl’s parents to take her out of the healer’s care and bring her to the health center. She made a complete recovery and is now in an apprenticeship. This happened without any negative impacts on the collaboration of traditional healers with community members.”

The social science team observed that as decentralized BU care removed many of the previous obstacles to seeking biomedical treatment for chronic wounds, community members were far more willing to consider this care option. Healers in Ouinhi who joined support groups recognized this as well. They found that participation in support groups and outreach programs added to their own prestige instead of diminishing it. Participation in a successful campaign reinforced their standing and trustworthiness in the public eye.

### Patient satisfaction with decentralized care

During interviews, patients identified a number of factors that made them more receptive to seeking decentralized care for BU than undergoing surgery, even when surgical care is free and offered by a hospital with an excellent reputation. The fear of surgery, grafting, and amputation, and the strong general association of CSNG with these practices, has long been a major factor in patient delay in the region [12, 25, 26]. The vivid image of amputation, kept fresh in the popular imagination by living examples, inspired particular terror especially among children. Grafting is also seen as a horrific practice because it involves the cutting of healthy flesh, a logic that does not translate well into local understandings of treatment and is generally regarded as perverse [12, 27, 28]. Community members often joke that when one goes to CSNG they go in with one wound and end up with two (due to grafting); the center is referred to in the communities by the name “*e kan bo tren*”, “they cut and attach”. Another common way of referring to the hospital is also telling: it is referred to as a prison, an image associated with the indeterminacy of one’s duration of stay.

Patients treated at Tohoue spoke of being very satisfied with decentralized care for three major reasons. First, surgery was avoided and this reduced their fear of being treated. Second, they had a clearer idea of how long treatment would last and they were better apprised of how their treatment was progressing. Indeterminacy was identified as a significant concern for households of those being treated for BU and linked to social displacement. Hospitalization imposes a significant economic challenge on households living on the margin through indirect and opportunity costs, even when most direct costs of hospital care are covered [29–31]. Patient and guardian displacement has serious repercussions for the household production of health [32] and requires securing replacement labor for agricultural and domestic tasks. Displacement is also psychologically wrenching, and a source of tremendous anxiety for patients and their care providers [7]. When the duration of displacement is uncertain, long-term planning for patients, guardians, and households is all but impossible. Interviews with patients revealed that a major reason for the popularity of decentralized care was that it offers a more clearly delineated source of treatment duration, enabling household activity and resource planning.

The following case vignette aptly illustrates why decentralized care appealed to a household that recognized that a family member had a chronic wound that required treatment, but were reluctant to seek care at CSNG hospital. The story involves a 38-year-old mother of three who chose to try decentralized care after seeking general wound care for her daughter at a clinic and hospital.

“When I first noticed the wound on my daughter, I brought her to the district hospital right away, but after a few days of care without any sign of improvement, I decided to try to treat the wound at home and hoped it would get better with time. But I was really worried. I was saying to myself that if home treatment doesn’t work I might have to take her to CSNG, which would entail all kinds of expenses and constraints. I was afraid. Just then, there was an outreach program on Buruli ulcer in my village. From the education provided, I understood things much better. During the program, a village volunteer informed us that from now on, free wound treatment was available at the local health center in Tohoue. That’s why I brought my child to the health center. She received treatment there for three months, and was completely healed. I really appreciated the availability of treatment in Tohoue. I was able to see my child healed in a short time and with hardly any expense. The other thing I really appreciated is that during treatment, she was able to continue going to school just as before. Every morning her daddy or I, depending on who was available, would take her to the clinic for bandaging and injection, and after receiving care she went to school and the rest of us could go back to our respective activities. This was a huge support for us, and a great relief.”

The informant, like several others interviewed, also commented on her interactions with the nurse in Tohoue, stating that he addressed her concerns and treated her with respect (“At the health center in Tohoue, the patient is treated with respect and love; the head nurse laughs with everyone”) (Fig 3B). The impact evaluation revealed that the nurse’s close connection to the community and empathy for patients were major factors contributing to the popularity of decentralized care.

## Discussion

Eight research questions were presented at the outset of the paper. We briefly address each here:

### Did the pilot intervention result in the identification and treatment of cases of BU that may not have come for surgical treatment following the centralized care model?

We have provided quantitative and qualitative evidence of Category I and early stage Category II BU cases being treated as a result of the combination of outreach education and the provision of decentralized care. Few cases of Category I BU are treated at CSNG hospital (2% compared to 12% at national level) and no cases of BU were treated at Tohoue clinic prior to the pilot project. Following outreach programs, 53 suspected BU cases were screened at the clinic and 77% of all confirmed cases were Category I or early stage Category II BU. This finding demonstrates the importance of community-based interventions and decentralized care in the control of BU [8, 14], and is all the more compelling because of the strength and diligence of CSNG’s surveillance program for case detection for many years prior to the intervention.

### How will the community respond to the innovative education program, and will it lead to cases of self-referral?

We found strong community support for the outreach program, both for its innovative educational approach and for the content of its messages. In particular, community members found the images that accompanied the text and oral presentation served as a useful mnemonic for accurate recall. Experienced volunteers and health staff who had been trained in earlier forms of BU outreach education expressed a preference for the new pedagogy as more dynamic and accessible. Self-referral rates after outreach were high.

### Will the community accept decentralized care and adhere to the antibiotic protocols?

The provision of free decentralized care eliminated or considerably reduced the primary constraints to seeking centralized treatment noted by patients who had delayed seeking care for BU. These constraints included practical and economic difficulties in sustaining prolonged displacement, the disruption of daily activities and fear of surgery and possible amputation. Community members were highly receptive to the idea of decentralized care, as evidenced by the number of cases arriving at the clinics after outreach programs. Patients who were confirmed as having Category I and II BU all elected to receive decentralized care, and all of them successfully completed the full course of antibiotic treatment.

### How did decentralized care and the outreach program affect women’s healthcare- seeking behavior?

One of the most significant impacts of the intervention was that women no longer needed to consult husbands (or other men in positions of authority) about attending clinics for BU treatment for themselves or their children. In Ouinhi, it is normative for women to consult men before traveling to a clinic. If men are not available, this can result in significant treatment delay and, in the case of a disease like BU requiring weeks of outpatient treatment, treatment non-adherence. Notably if a woman is required to travel to a clinic daily, this may place her in a position of social risk to rumor about her moral identity. As a result of the outreach education, BU became the exception to this general rule. Community members came to understand the necessity for continuous treatment at the clinic. Women attending clinics did not report feeling vulnerable to social critique if they went to the clinic in the absence of their husbands, nor subject to social stigma for their movements outside their households or villages. We observed this change occur in real time in Ouinhi.

### Does the creation of social support groups add value for case detection, patient treatment adherence, and volunteer motivation?

Nearly half (45%) of the chronic ulcers treated at Tohoue were referred to the clinic by members of community support groups. Support groups also proved effective in approaching family members to negotiate the transfer of patients from traditional medicine to biomedical care, and served a valuable role in following up with cases that dropped out of therapy or were non-adherent. All such cases completed treatment once contacted by support groups. Support group members received praise for their role in outreach activities by government health staff during monthly meetings held at local clinics. Health staff interviewed during the impact evaluation appreciated the support group model and hoped to leverage BU groups to assist them in implementing health programs beyond Buruli ulcer.

When designing the pilot intervention, the team was attentive to the many tasks and responsibilities of community volunteers. In another district (Zé) of Benin, social science team members found that when linear programs like BU establish separate cadres of volunteers, jealousy over resources and confusion over roles occurs between volunteers associated with different groups. During the pilot project, existing volunteers were placed within BU support groups comprised of many different kinds of stakeholders. Support group members saw their role as assisting health volunteers perform their many tasks, and tensions among members was not reported. Volunteers very much appreciated this assistance.

### Will the treatment of all chronic ulcers at local clinics increase the reputation of the clinic and community satisfaction with the BU decentralized care program?

The treatment of all chronic ulcers free of charge reduced the chance of clinic staff being accused of favoritism by a population that little understands the reasoning behind vertical programs, especially programs that do not involve contagious diseases. Assurance that wound treatment costs would be covered regardless of diagnosis was a major factor in convincing people who were hesitant to consult the clinic before to do so now. The reputation of the Tohoue clinic in the community was positive to begin with, but wound care was not seen as a service routinely provided given that chronic wounds were generally referred. Introducing chronic wound care increased the clinic’s reputation and the staff were soon recognized as having expertise. Patients interviewed about their treatment at the clinic were very satisfied with the care they received and have become the best advocates of decentralization care being piloted. Some have volunteered to present testimonials of their care at outreach meetings.

### How will clinic staff respond to an expanded scope of wound care management, and what will be the impacts of better feedback to clinicians about the accuracy and outcome of their referrals?

Practical training in wound care was well received by clinic staff. Monitoring by staff from Allada found that training resulted in greater self-confidence to treat, sound clinical practice, and appropriate referrals to hospital in keeping with established treatment guidelines. Ongoing feedback about referrals was much appreciated by clinic staff and constitutes a form of continuing education that should increase efficiency and reduce costs for the health system [33].

### Can a good working relationship between the decentralized and centralized BU care programs be established?

For more than a decade it was not possible to set up decentralized BU care in the Zou department. The pilot intervention demonstrated that this is possible when introduced as a win-win situation for the CSNG hospital and local clinics. The hospital offering centralized care is happy to have Category I BU cases and non- BU chronic ulcers treated at local clinics, freeing them to attend to and use their resources for more serious cases. The relationship between the CSNG hospital and local clinics participating in decentralized care is still fragile, however, and cannot yet be described as collaborative.

### Challenges

There are four challenges that will need to be faced if decentralized treatment for BU is to go to scale in the Zou department and be sustainable. First, it will be important to mainstream the programs such that wound care becomes part of the routine scope of work for the clinic nurse. Next, wound care outreach and clinical care will need to be coordinated, and the program locally supervised. The experience gained by the national Buruli ulcer program through the implementation of this pilot program must be capitalized upon, so that this program can serve as the technical and administrative foundation for an integrated wound management program at the regional and health district level.

A second challenge is the attrition of trained staff due to re-assignment. New health staff will continually need to be trained in wound care. It is worth looking into whether this can be done through short workshops followed by field apprenticeships with experienced health staff who have demonstrated good wound care skills.

A third challenge will be keeping BU support groups, health volunteers and clinic staff motivated. Resources are necessary for members of support groups to accompany suspected cases of BU to the clinic, as some community members feel reluctant to visit clinics without a person familiar with the clinic to help support them during an initial visit. Volunteers who accompany suspected patients for screening require travel funds. Motivation for support group members will also need to be provided if they are charged with participating in an integrated NTD and wound care program. The importance of face-to-face meetings for sustaining collaboration has been noted elsewhere in Africa [34]. The motivation of clinic staff also must be taken into account. If asked to increase their workload to include wound care, they need to be provided with the resources to do so, and receive some form of incentive or recognition [34]. Research is currently under way to determine what kinds of motivations are effective and feasible for volunteers and clinic staff.

A fourth challenge is the need for wound care outreach education as a complement to the BU outreach programs currently being conducted. As pointed out by clinic staff, education about wound care and skin disease management is needed so clinics are not swamped with cases that may be managed at home, or cases managed in the clinic that require follow-up care at home associated with wound hygiene and scar management.

The intervention of the project had a very positive impact on Tohoue clinic attendance, which has notably increased since the outreach program and introduction of decentralized wound management. According to the health workers, the demand for medical care had significantly increased. Three fundamental factors have affected the attendance of the health center: the effect of the awareness-raising sessions, proximity of care, and free medical care for all chronic wounds.

The awareness-raising sessions allowed people to have information about the disease (its causes, manifestations and treatment), but also to become aware of the sequelae and social consequences that it could generate. As a result, the population was notably more prompt to resort to the center for any BU-like sign or symptom. The proximity of care was also described as one of the motivating factors in the search for medical treatment in the population, since patients no longer need to travel more than thirty kilometers for medical care, and households no longer need to worry about providing caregivers for patients in hospital. The free medical treatment was also one of the main reasons why they systematically go to the health center in case of problem.

“The decentralized care is beneficial for our community. Indeed, in the past, to bring a relative to Zangnanado, we had to find the resources for feeding them while in hospital and arranging for transport, not to mention the fact that the patient will be isolated from the community; all these reasons make people not adhere to treatment. This decentralized care had helped to limit the advanced cases, because people referred early; it saved us from traveling and abandoning our activities.” (Community member)

As the clinic’s prestige has risen, a concomitant decrease in the use of traditional healers has been observed in the community.

“People are going less and less to the traditional healers. They are increasingly deciding to go to the health center instead of going to the healers.” (Health worker)

### Limitations of the study

Staff from three clinics were trained in BU and chronic wound care in this pilot study. However, only one clinic was fully functional during the entire intervention period. Staff from the other clinics were assigned elsewhere during the study. When new staff were then sent to short trainings in Lalo and Allada they were not as motivated as staff initially trained in longer, more hands-on and comprehensive courses. These staff preferred to refer cases to Tohoue clinic. Staff transfer limited our ability to learn from a larger sample of clinics. Another limitation was the inability of CSNG to offer technical supervision of wound care during the intervention. Lack of staff prevented the local hospital from offering supervision, and this had to be offered by the staff of CDTUB Allada. To ensure sustainability, if the intervention goes to scale, health district authorities will need to support monitoring activities locally.

### Conclusion

Four lessons learned from this successful community outreach and pilot decentralization care program for BU may be highlighted as having relevance for other health interventions.

First, the dramatic increase in BU case detection in a region already having proactive case detection activities associated with free centralized care illustrates the importance of more accessible care that does not engender indirect, social and opportunity costs associated with displacement. Secondly, positive reception of the program reveals the importance of formative, action-oriented research in guiding program design. Thirdly, the program’s success suggests significant contributions of culturally sensitive outreach education that is responsive to questions, and the role of community volunteer groups in detection, negotiating participation, and providing support to patients and households.

The pilot project also demonstrates the added value of integrated wound management for the control of neglected tropical skin diseases [35]. The importance of integrated prevention and care models has long been recognized as integral to strengthening health systems and improving quality and sustainability in health care [35–38]. This program not only produced improved outcomes for Benin’s NTD program, but also addressed the local need for community wound care as basic to primary care. It also demonstrated that community-based programs which bring care closer to the people increase the motivation of community volunteers and health staff serving in peripheral health centers [39]. The reputation and status of both is enhanced. Based on the success of this project a larger community-based wound care pilot project is now under way in the region. This new project will explore in greater detail the costs of bringing integrated neglected skin diseases/ chronic wound care provision to scale along with community outreach programs on home-based wound care. As decentralized BU treatment adopts oral therapy (with rifampicin and clarithromycin) for the treatment of Category I cases, appropriate home based wound care management will become all the more important.
